# Editorial: Development and Application of Clostridia as Microbial Cell-Factories for Biofuels and Biochemicals Production

**DOI:** 10.3389/fbioe.2021.831135

**Published:** 2022-01-11

**Authors:** Hongxin Fu, Shang-Tian Yang

**Affiliations:** ^1^ Guangdong Key Laboratory of Fermentation and Enzyme Engineering, School of Biology and Biological Engineering, South China University of Technology, Guangzhou, China; ^2^ William G. Lowrie Department of Chemical and Biomolecular Engineering, The Ohio State University, Columbus, OH, United States

**Keywords:** clostridia, butanol, butyric acid, isobutanol, 1,3-butanediol (1,3-BDO), biofilm, short- and medium-chain ester, metabolic engineering

Clostridia are Gram-positive, spore-forming, obligate anaerobic bacteria with versatile substrate utilization and metabolite production capabilities (Xue et al., 2017). The exploitation of clostridia for large-scale production of commodity chemicals can be dated back to 100 years ago with solventogenic clostridia in acetone-butanol-ethanol (ABE) fermentation, which had fallen out of favor since the establishment of more economical petrochemical processes (Cheng et al., 2019a). However, the necessity of sustainable development has renewed interest in the production of biofuels and biochemicals from abundant renewable biomass. In recent years, several *Clostridium* species, including conventional solventogenic clostridia such as *C. acetobutylicum* and *C. beijerinckii*, cellulolytic clostridia like *C. thermocellum*, *C. cellulolyticum* and *C. cellulovorans* (Yang et al., 2015), acetogens such as *C. formicoaceticum* (Bao et al., 2019) and *C. carboxidivorans* (Cheng et al., 2019b), and acidogens including *C. tyrobutyricum* (Li et al., 2019; Bao et al., 2020; Fu et al., 2021) *and C. kluyveri*, have garnered immense interests in the field of industrial biotechnology for their abilities to produce various chemicals and biofuels from low-cost agricultural residues and industrial wastes.

The high toxicity of fermentative products (such as butanol and butyrate) is a drawback limiting Clostridia for industrial applications. Random chemical mutagenesis aiming at increasing butanol efflux capacity was used to improve butanol tolerance of *C. beijerinckii* (Vasylkivska et al.). Mutant strains obtained by different approaches behaved differently in terms of efflux pump substrates (butanol, ethanol, ethidium bromide and antibiotics) tolerance and metabolites production. The best mutant obtained with ethidium bromide for mutagenesis and selection showed a 127% improvement in butanol tolerance. The genomes of various mutant strains were sequenced and analyzed, and the results indicated that the improved butanol tolerance was attributed to mutations in genes related to stress responses (chemotaxis, autolysis or changes in cell membrane structure) and efflux pump regulators. This study confirmed the importance of efflux in butanol stress and provided new gene targets for rational strain engineering. On another hand, Gao et al. screened 5 *C. acetobutylicum* mutants through carbon ion beam irradiation, which had advantages of excellent biological effectiveness and dose conformity over traditional mutation methods. The mutant Y217 showed enhanced butanol tolerance and production of 13.67 g/L (vs. 9.77 g/L for the control), which was attributed to its ability to maintain cell membrane integrity and permeability under butanol stress. As strain mutagenesis and screening is a time-consuming process, Cao et al. developed and verified non-structured mathematical models which could reflect strain growth, glucose consumption, and butyric acid production of ^12^C^6+^ irradiation-mutation strain of *C. tyrobutyricum*.

Although clostridia possess the ability to ferment a broad range of substrates, carbon catabolite repression (CCR), which usually happens when mixed sugars (glucose and non-glucose substrates) are present in the fermentation medium, is limiting their uses of lignocellulosic biomass hydrolysates as substrates (Fu et al., 2017). Although catabolite control protein A (CcpA) knockout resulted in simultaneous utilization of glucose and xylose in *C. acetobutylicum*, some negative influences were observed due to the multiple roles of CcpA (Wu et al., 2015). To solve this problem, Ujor et al. explored a ribozyme-mediated approach to downregulate CcpA in *C. beijerinckii*. The expression of CcpA-/DisA (encoding DNA integrity scanning protein A)-specific M1 RNA-based ribozyme led to obvious decreases in CcpA/DisA mRNA levels and modest increase in mixed sugars utilization and ABE production compared to the control. This study demonstrated that DisA played an important role in regulating solvent production and the feasibility of using ribozyme-mediated approach for gene knockdown in *C. beijerinckii.*

Isobutanol is an important platform chemical widely used in food, chemical, biofuel and pharmaceutical industries. The market of bio-based isobutanol reached $1 billion in 2019 and is expected to rise significantly in the near future. Weitz et al.introduced two different isobutanol synthesis pathways, ketoisovalerate ferredoxin oxidoreductase (Kor) and ketoisovalerate decarboxylase (Kivd), into two acetogenic bacteria, *Clostridium ljungdahlii* and *Acetobacterium woodii*, and evaluated their effects on isobutanol production under both heterotrophic and autotrophic conditions. When syngas was used as the substrate, the engineered *C. ljungdahlii* produced 0.4 and 1 mM isobutanol without and with ketoisovalerate addition via the Kivd pathway, which was better than the results (0 and 0.2 mM isobutanol) obtained via the Kor pathway. To identify the bottlenecks of autotrophic isobutanol production, syngas-based batch cultivation together with metabolic profiling and flux balance analysis were performed for various *C. ljungdahlii* strains (Hermann et al.), and the results indicated that further work could be focused on improving the activities of key enzymes and changing their coenzyme specificity or supply. The chirally pure (R)-1,3-butanediol (BDO) is used for fragrances, insecticides and as precursor molecules for penem and carbapenem antibiotic synthesis. Grosse-Honebrink et al. expressed acetoacetyl-CoA reductase gene *phaB* from *Cupriavidus necator* in *C. saccharoperbutylacetonicum* and optimized the heterologous pathway at transcriptional (promoters and gene expression methods optimization), translational (codon optimization), enzyme (point mutations), and population (medium optimization) levels for (R)-1,3-BDO production from glucose. The optimized mutant produced 1.8 g/L (R)-1,3-BDO, a 217% increase compared to the control. A higher concentration could be achieved by further optimizing the fermentation process.

*C. acetobutylicum* has long been and extensively used in industrial production of acetone, butanol, and ethanol in ABE fermentation, but little attention has been paid to its biofilm. Zhang et al. summarized the cell physiological changes, extracellular matrix components, production advantages, influencing factors and regulatory genes of *C. acetobutylicum* biofilm, which provided valuable insights into its molecular basis useful for developing efficient biofilm processes. Clostridia can produce a variety of organic acids and alcohols, and thus are promising whole cell biocatalysts for short- and medium-chain esters. Wang et al. reviewed the advances in ester production by Clostridia including *in vitro* lipase catalysis and *in vivo* acyltransferase reaction. In addition, the potential of several Clostridia and clostridial consortia which can utilize cheap substrates such as industrial waste gas and lignocellulose for bio-ester production and the recent development of synthetic biology in clostridial chassis development were also discussed. Furthermore, cellulolytic Clostridia in a constructed cellulolytic microflora played an important role in anaerobic co-digestion of pig manure and rice straw for methane production (Zhong et al.).

In summary, the original research and review papers published in this Research Topic provide valuable information on the selection and construction of robust and novel *Clostridium* strains for biofuels and biochemicals production from renewable resources, which will contribute to the further development of Clostridia as microbial cell-factories for industrial applications.
